# *Daphnia magna* Exudates Impact Physiological and Metabolic Changes in *Microcystis aeruginosa*

**DOI:** 10.3390/toxins11070421

**Published:** 2019-07-19

**Authors:** Gorenka Bojadzija Savic, Christine Edwards, Enora Briand, Linda Lawton, Claudia Wiegand, Myriam Bormans

**Affiliations:** 1 Univ Rennes, CNRS, ECOBIO-UMR 6553, F-35000 Rennes, France; 2 School of Pharmacy and Life Sciences, Robert Gordon University, Aberdeen AB10 7GJ, UK; 3 IFREMER, Phycotoxins Laboratory, F-44311 Nantes, France

**Keywords:** cyanobacteria, secondary metabolites, PCC7806, toxic, mutant, infochemicals

## Abstract

While the intracellular function of many toxic and bioactive cyanobacterial metabolites is not yet known, microcystins have been suggested to have a protective role in the cyanobacterial metabolism, giving advantage to toxic over nontoxic strains under stress conditions. The zooplankton grazer *Daphnia* reduce cyanobacterial dominance until a certain density, which may be supported by *Daphnia* exudates, affecting the cyanobacterial physiological state and metabolites’ production. Therefore, we hypothesized that *D. magna* spent medium will impact the production of cyanobacterial bioactive metabolites and affect cyanobacterial photosynthetic activity in the nontoxic, but not the toxic strain. Microcystin (MC-LR and des-MC-LR) producing *M. aeruginosa* PCC7806 and its non-microcystin producing mutant were exposed to spent media of different *D. magna* densities and culture durations. *D. magna* spent medium of the highest density (200/L) cultivated for the shortest time (24 h) provoked the strongest effect. *D.magna* spent medium negatively impacted the photosynthetic activity of *M. aeruginosa* PCC7806, as well as the dynamics of intracellular and extracellular cyanobacterial metabolites, while its mutant was unaffected. In the presence of *Daphnia* medium, microcystin does not appear to have a protective role for the strain. On the contrary, extracellular cyanopeptolin A increased in *M. aeruginosa* PCC7806 although the potential anti-grazing role of this compound would require further studies.

## 1. Introduction

Cyanobacterial blooms can occur in freshwater ecosystems due to eutrophication, often producing toxic and bioactive secondary metabolites that can affect the life traits of all organisms of the ecosystem [1]. At the same time, zooplankton graze on phytoplankton, including cyanobacteria, and as such it has been suggested that *Daphnia* are able to reduce cyanobacterial dominance in aquatic ecosystems up to a certain density and toxicity [2,3,4]. Cyanobacteria can impair planktivorous zooplankton by several mechanisms including the action of toxic metabolites or compounds inhibiting digestive enzymes, their nutritional inadequacy, or simply the mechanical interference during feeding [5,6]. Consequences are reduced growth, survival, and reproduction [7,8,9]. *Microcystis* is a widespread genus of freshwater cyanobacteria, that produces microcystins (MCs), the most commonly investigated cyanotoxins, with over 250 variants [10]. The toxicity of MC is reflected in a wide range of organisms including mammals, plants, and zooplankton through the inhibition of serine-threonine protein phosphatase typ 1 and 2A [11,12]. Blocking the protein phosphatases typ 1 and 2A moves the critical balance between phosphorylated and dephosphorylated proteins towards the hyperphosphorylated state with consequences for cellular metabolism including energy allocation, gene expression and translation, and cytoskeletal components [13]. Oxidative stress is another mechanism, which impairs the cellular function on protein, lipid including membranes, and the nucleic acid level [14]. If not detoxified, damages occur from cellular to organism level, with death as the ultimate consequence [15]. Cyanobacteria produce a wide range of secondary metabolites such as non-ribosomal peptides, polyketides, ribosomal peptides, alkaloids, and isoprenoids [16]. The complexity and dynamics of biotic and abiotic factors (availability of nutrients, light, pH, temperature) may influence cyanobacterial growth and production of cyanobacterial secondary metabolites (cyanotoxins and other compounds) [1,17]. Additionally, the biosynthesis of secondary metabolites occurs at the expense of energy [18], therefore, the understanding of metabolites or pathways that trigger cyanobacterial secondary metabolites production is of high importance for their management in aquatic environments. 

It has been suggested that secondary metabolites are produced as a response to rapid changes in the environment and may have a role as a competitive advantage [1]. Potential functions of microcystin such as an “iron-scavenging” molecule [19,20], intracellular protection [21,22,23], anti-grazing role [5,24,25,26,27] have been proposed, however, understanding their ecological role is still challenging [28]. MC-producing species have been assumed to have a higher chance of survival compared to nontoxic species [1,29,30]. Recent research raises the possibility that microcystins may play a role in enhancing the ability of surviving oxidative stress [1,31]. The increased production of intracellular MC under conditions of stress [32] allowed MC-producing strains to cope better with oxidative stress over non-toxic strain [21,30,33]. However, other findings show opposite results: The nontoxic *M. aeruginosa* PCC7806 mutant was favored over its toxic wild type when exposed to prolonged oxidative stress caused by hydrogen peroxide [34]. 

Most reported studies on MC production explore how abiotic factors influence toxin production, while only a few studies aim to understand the impact of biotic factors (mainly phytoplankton competition and zooplankton grazing) as MCs triggers in *Microcystis* [26,35,36,37]. The role of cyanobacterial metabolites could be a defense mechanism against predators [38], where toxic species have a higher chance of survival compared to nontoxic species [1,39]. Conversely, molecular evidence clearly shows that genes responsible for toxin production dates back to before the reported existence of more complex organisms [40]. Therefore, cyanobacterial secondary metabolites might have evolved their role over time as a chemical defense against predators [29,41]. However, triggers for the production of cyanobacterial metabolites in the presence of grazers and their role is still scarce [42]. While some studies show that *Daphnia* via infochemicals induce MC production [25,43] others suggest that *Daphnia* infochemicals have a weak influence on MC dynamics [44], or even inhibit its production [45]. Although it has been suggested that *Daphnia* via infochemicals can impact cyanobacterial metabolites dynamics [25,38,46,47], their chemical structures remain unknown, due to challenging detection and isolation [48]. 

In addition to MCs, *Microcystis* produces a range of peptides, such as aeruginosins, anabaenopeptins, cyanobactins, cyanopeptolins, microginins, and microviridins [49], out of which several negatively influence zooplankton population [50,51,52,53,54]. The growth of *Daphnia magna* was impaired when exposed to nontoxic mutant of *M. aeruginosa* PCC7806 *mcy-* in comparison with the wild-type strain, suggesting the involvement of other secondary metabolites that have negative impact on *Daphnia* feeding [55]. Sadler and von Elert (2014) suggested that cyanopeptolins and aerucyclamides could have a potential role against *D. magna* grazing, as their productions were elevated in the presence of *D. magna* [26]. However, the role of metabolites, as well as the triggers for their production remain unclear [36]. There is no data addressing microviridins and aeruginosins production induced by the presence of *Daphnia* and despite their trypsin inhibition capacity [56], it has yet to be understood, if their role could involve active anti-grazing activities. 

This study investigates whether the presence of *D. magna* infochemicals (via spent medium (DM)) affects secondary metabolites production, the physiological state in the *M. aeruginosa* PCC7806 (MC+), and its mutant *M. aeruginosa* PCC7806 *mcy-* (MC-). We hypothesize that the presence of *D. magna* infochemicals will: (a) Induce the production of microcystins and other cyanobacterial bioactive compounds in both strains and (b) have negative impact on photosynthetic activity in *M. aeruginosa* PCC7806 mutant, but not *M. aeruginosa* PCC7806.

## 2. Results

### 2.1. Photosynthetic Activity

**Figure 1:**
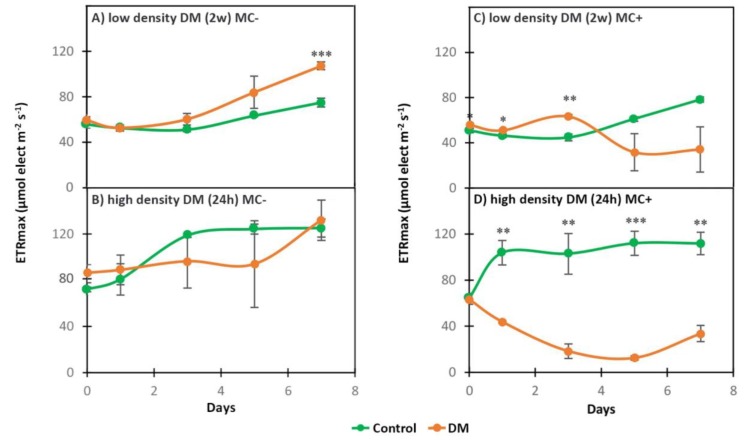
Photosynthetic activity of MC- and MC+ when cultured in different *D. magna* spent medium (DM) and cultured in BG11 (Control): (**A**) low density DM (2w) MC− (**B**) high density DM (24 h) MC- (**C**) low density DM (2w) MC+ (**D**) high density DM (24 h) MC+. * (*p* < 0.05), ** (*p* < 0.01), *** (*p* < 0.001) *t*-test.

The photosynthetic activity of the strains was measured through the maximum electron transport rate ETRmax. When exposed to spent medium from 75 all age individuals of *D. magna* /L raised for two weeks (low density DM (2w)), MC- was not negatively affected, on the contrary, it remained in good physiological state during seven days of exposure (Figure 1A, Table 1). However, in the MC+ strain the photosynthetic activity showed a non-statistically significant decrease after being initially significantly higher during the first three days of exposure and did not recover compared to the control (Figure 1C, Table 1). Then, both strains were exposed to a medium in which a higher density of *Daphnia* (200 all age individuals of *D. magna* /L) was cultured for 24 h (high density DM (24 h)). This exposure had contrasting effects on the strains: The photosynthetic activity of the MC- was high during the entire experiment in the exposed cells as well as in the control (Figure 1B, Table 1), whereas the MC+ strain suffered from a statistically significant decrease of photosynthetic activity during the whole experiment compared to the control (Figure 1D, Table 1).

Photosynthetic activity was also compared between the MC+ and MC- strains in those exposures showing differences between treatment and control (low density DM (2w) and high density DM (24 h), Table 1). In the low density DM (2w) exposure, control MC+ and control MC- showed no significant difference during the experiment, except on the first day. Photosynthetic activity of the low density DM (2w) treatments was not significantly different between the strains during the first three days of the experiment, but it significantly decreased in MC+ on day 5 and 7 compared MC- exposed to low density DM (2w) (Table 1). In the high density DM (24 h) exposure, there was no statistically significant difference between control MC+ and control MC-. However, photosynthetic activity in MC+ exposed to high density DM (24 h) significantly decreased during the whole experiment (except on day 5, owing to a high variability in MC-) compared to MC- exposed to high density DM (24 h) (Table 1).

### 2.2. Dynamics of Intracellular and Extracellular Metabolites in MC+ and MC-

#### 2.2.1. Metabolic Profiles of MC+ and MC– 

**Figure 2:**
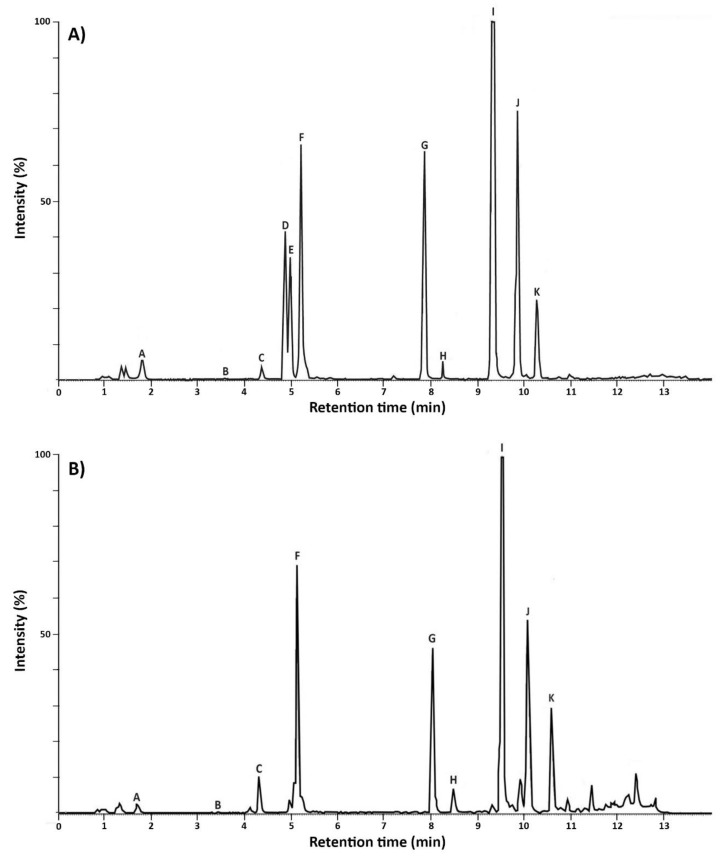
Secondary metabolites detected in MC+ (**A**) and MC- (**B**). A aeruginosin 684, B cyanopeptolin B, C aeruginosin 602, D des-MC-LR, E MC-LR, F cyanopeptolin A, G aerucyclamide D, H cyanopeptolin 963A, I aerucyclamide A, J aerucyclamide C, K aerucyclamide B.

Eleven metabolites produced by MC+ (Figure 2A) were detected and nine metabolites produced by MC-(Figure 2B). MC+ produced microcystins (LR and des-MC-LR), cyanopeptolins (963A, A, and B), aerucyclamides (A, B, C, and D) and aeruginosins (684 and 602) (Figure 2A). MC- produced cyanopeptolins (963A, A and B), aerucyclamides (A, B, C, and D) and aeruginosins (684 and 602) (Figure 2B).

#### 2.2.2. Dynamics of Intracellular and Extracellular Metabolites in MC+ and MC- When Exposed to Low Density DM (2w)

**Figure 3:**
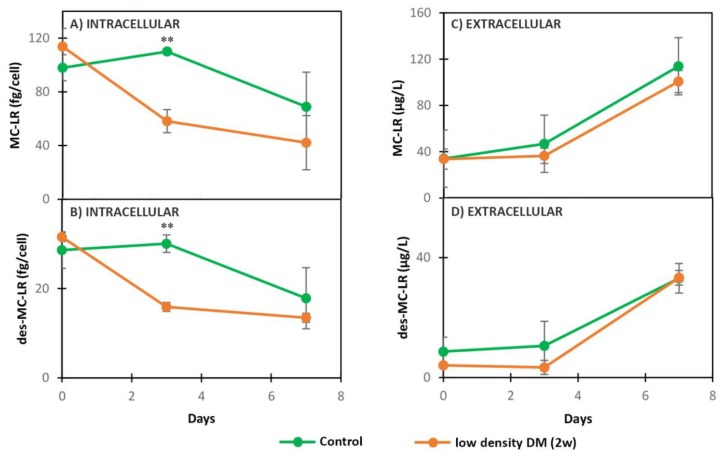
Dynamics of intracellular and extracellular metabolites in MC+ when exposed to low density DM (2w) and grown in BG11 (Control): (**A**) intracellular MC-LR, (**B**) intracellular des-MC-LR, (**C**) extracellular MC-LR, (**D**) extracellular des-MC-LR. ** (*p* < 0.01) *t*-test.

**Figure 4:**
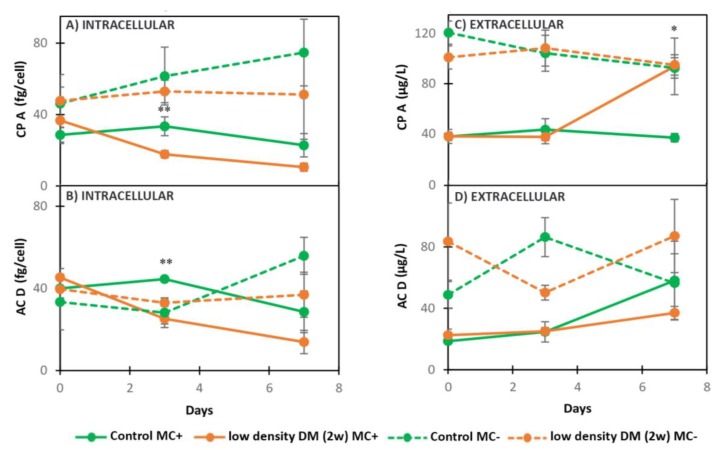
Dynamics of intracellular and extracellular metabolites in MC+ and MC- when exposed to low density DM (2w): *M. aeruginosa* PCC7806 (low density DM (2w) MC+) and *M. aeruginosa* PCC7806 *mcy-* (low density DM (2w) MC-) and when grown in BG11: *M. aeruginosa* PCC7806 (Control MC+) and *M. aeruginosa* PCC7806 *mcy-* (Control MC-). (**A**) intracellular CP A, (**B**) intracellular AC D, (**C**) extracellular CP A, (**D**) extracellular AC D. * (*p* < 0.05), ** (*p* < 0.01) *t*-test.

Low density DM (2w) significantly decreased the concentration of intracellular MC-LR and des-MC-LR at the second day of exposure in comparison with the control (Figure 3A,B, Table 2). The dynamics of extracellular MC-LR and des-MC-LR were not significantly different between the control and treated cells during the experiment in MC+ (Figure 3C,D, Table 2). Intracellular AC D and CP A produced by MC+ followed a decreasing trend when exposed to low density DM (2w), with significant difference on day 3 in comparison with the control (Figure 4A,B, Table 2). While the export of AC D produced by MC+ did not change significantly in the treatment compared with the control (Figure 4D, Table 2), CP A was excreted in significantly higher amounts in the presence of low density DM (2w) in MC+ on day 7 of the experiment in comparison with the control (Figure 4C, Table 2). Neither the intracellular nor extracellular dynamics of CP A and AC D produced in MC- were statistically different when exposed to low density DM (2w) compared to the control (Figure 4A–D, Table 2). 

The concentration of intracellular and extracellular CP A and AC D was compared between MC+ and MC- in the low density DM (2w) exposure experiment (Table 2). The intracellular concentration of CP A and AC D was significantly higher in the control MC- when compared with the control MC+ on day 3 and 7. The concentration of intracellular CP A was significantly higher in low density DM (2w) MC- compared to low density DM (2w) MC+ on day 3, but not on day 0 and 7. There was no significant difference in AC D intracellular concentration in low density DM (2w) MC+ compared to low density DM (2w) MC- during the whole experiment. The concentration of extracellular CP A significantly increased in control MC- compared to control MC+ on day 3 and 7. The amount of extracellular AC D was significantly higher on day 0 and 3 in control MC- compared to control MC+, while there was no difference on day 7. When MC+ and MC- were exposed to low density DM (2w), the concentration of extracellular CP A was significantly lower in low density DM (2w) MC+ compared to low density DM (2w) MC- on day 0 and 3 of the experiment, while there was no statistical significance on day 7. The concentration of extracellular AC D was significantly lower in low density DM (2w) MC+ compared with low density DM (2w) MC- on day 3, while on day 0 and 7 there was no statistically significant difference (Table 2).

#### 2.2.3. Dynamics of Intracellular and Extracellular Metabolites in MC+ When Exposed to High Density DM (24 h)

**Figure 5:**
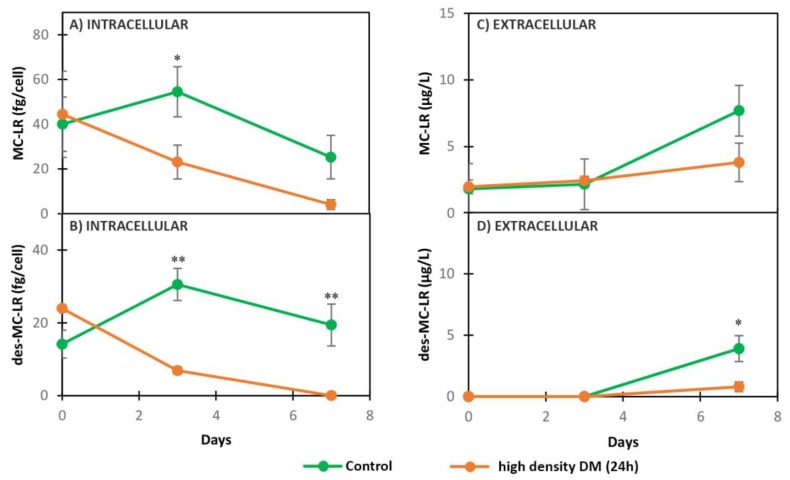
Dynamics of intracellular and extracellular metabolites in MC+ when exposed to high density DM (24 h) and grown in BG11 (Control): (**A**) Intracellular MC-LR, (**B**) intracellular des-MC-LR, (**C**) extracellular MC-LR, (**D**) extracellular des-MC-LR. * (*p* < 0.05), ** (*p* < 0.01) *t*-test.

When exposed to high density DM (24 h), the concentrations of intracellular MC-LR and des-MC-LR by MC+ were reduced at three days of exposure, compared with the control, and did not recover during the experiment (Figure 5A,B, Table 3). Reduction of intracellular metabolites was followed by a non-statistically significant lower excretion of MC-LR and significantly lower excretion of des-MC-LR in treatment compared to the control on day 7 (Figure 5C,D, Table 3). CP A and AC D were detected in both strains in control and 24 h DM treatment, however they were below the limit of quantification. Hence, unfortunately, no results on the CP A and AC D concentrations are available for this exposure. 

## 3. Discussion

MCs are by far the most commonly investigated toxins in cyanobacteria [57], but their role and regulation still remain unclear. While abiotic factors have been extensively studied, biotic factors including responses to grazing have received less attention. In this study, we compared the responses of both a MC-producing strain and its nontoxic mutant to *Daphnia magna* exudates via spent medium exposures in order to test the potential role of MC and/or other cyanopeptides as a response to the presence of grazers.

It is believed that most cyanobacterial species retained this pathway due to the potential protective role of MCs against oxidative stress, favoring the proliferation of MC-producing strains over nontoxic ones under stressful conditions [33]. Under growth limiting conditions of light, temperature, and nitrogen, MC-producing *Planktothrix agardhii* strains showed better fitness than nontoxic strains [30]. Similarly under growth limiting conditions of light and nitrogen, MC-producing *M. aeruginosa* showed better fitness than nontoxic strains [58]. A proteomic study showed that MC-producing *M. aeruginosa* species coped better with environmental changes that induced production of ROS than the nontoxic strain, hypothesizing possible functions of MC as a scavenger of free radicals [21]. Zilliges et al. (2011) revealed that if MC binds to proteins, it increases their stability, suggesting an important protective role of MC against stress [22]. 

The possibility of stress in cyanobacteria induced by grazers, such as *Daphnia* sp, has gained scientific interest. Most of the studies looked at the indirect effect of *Daphnia* on cyanobacteria used media in which *Daphnia* were cultivated for short periods of time, usually 24 h–48 h [25,26,38,46,59]. Effects of aged zooplankton medium on cyanobacteria are scarce in the literature, although the culturing of *Daphnia* for a longer duration, allowing the accumulation of *D. magna* metabolites over time, would be environmentally more relevant. As the spent medium of low density DM (1w) seemed to have no effect on both strains in this study, *D. magna* were cultivated for one more week in order to accumulate more metabolites that would potentially evoke an effect on cyanobacterial cell physiology. The photosynthetic activity (as ETRmax) is often measured as proxy of the physiological state of algal cells, as in this study. That parameter as a precursor of growth [58,60], indicates a poor physiological state from which the strain does not recover when ETRmax values are below 30, while values reaching 100 and above are associated with a healthy physiological state [58]. Contrary to our expectations, the toxic strain decreased its photosynthetic activity (ETRmax < 30), coping poorly with stress caused by low density DM (2w), while the mutant strain stayed in good physiological state during the entire experiment (seven days). These findings are supported by a recent study using the same strains, also discovering higher sensitivity of the toxic strain, when comparing responses of both strains to prolonged oxidative stress caused by hydrogen peroxide: Only the mutant was able to recover and regain initial photosynthetic yields [34]. As cyanobacterial reactions could be strain specific, different strain’s reactions are important to consider: When MC-producing *M. aeruginosa* strains Ch10 and UTEX LB2385 were exposed to five-day old *D.magna* spent medium, the content of chlorophyll also decreased, similar to our results [47]. 

In environments that promote oxidative stress MC binds to proteins [22,61], amongst others to the same target protein as thioredoxin [62] and possibly peroxiredoxin [34]. Binding of MC to these proteins will render them inactive, and upon loosing these antioxidant activities, the MC producing cells become more sensitive to oxidative stress [34]. Furthermore, the higher expression of genes regulating anti-oxidative enzymes in the nontoxic mutant, may favor the nontoxic mutant over the toxic strain when it comes to coping with high levels of oxidative stress [34]. 

Initial cyanobacterial density and concentration of *Daphnia* infochemicals influence the interaction between *Daphnia* and cyanobacteria [63]. In order to address this and avoid possible bias by a bacterial degradation of *Daphnia* metabolites, a culture of lower cyanobacterial cell density was exposed to high density DM (24 h). This experiment confirmed tendencies observed in the previous exposures by a drastically decreased photosynthetic activity in the toxic strain (ETRmax < 30), while the mutant stayed in good physiological state during the whole experiment. This suggests that the nontoxic mutant was coping better, compared with the toxic strain, when exposed to high density *D. magna* spent medium.

Depending on the density of *D. magna* and cultivation time, concentrations of intra- and extracellular MC-LR and des-MC-LR differed in the exposures: Whereas lower *D. magna* densities caused increasing tendencies of the toxins, higher *D. magna* densities and in particular those with short time for bacterial degradation of infochemicals caused a decrease. Similarly, *M. aeruginosa* increased MC production when exposed to increasing *D. magna* spent medium concentrations, suggesting an anti-grazer role triggered by zooplankton infochemicals [23], when only 50 adult *D. magna* were grown for four days per L of dechlorinated water of which 10–50% were mixed with the cyanobacterial culture. In addition, the presence of large cladocerans such as *D. cucullata* and *D. longispina* in the Sulejow Reservoir (Poland) positively correlated with the increase of MCs content in *Microcystis* [35]. Similarly, in the presence of *D. pulex*, the production of MC was significantly higher in *Microcystis* spp in all tested cyanobacterial (0.5–4.5 × 10^6^ cells/mL) and *D. pulex* (100–500 individuals/L) densities [27]. Different developmental stages of *Daphnia* can promote different production of MCs [25,35] because the same number of adults can possibly produce more, or other infochemicals than neonates, inducing higher MC production [43]. Although these studies support the hypothesis that a possible role of MC could be as anti-grazer defense [25,27,35], molecular evidence clearly shows that genes responsible for toxin production are much older than the existence of more complex organisms [40]. Furthermore, Kurmayer et al. 2016 [64] pointed out the presence and global distribution of both toxic and nontoxic cyanobacterial genotypes, suggesting their co-existence through evolution, as none of them were strongly favored by natural selection. Therefore, the potential role of cyanobacterial secondary metabolites as a defense mechanism against grazers, might be an adaptation they acquired over time [39,41]. However, not all studies support these results (e.g., [38,45]. Van Gremberghe et al. (2009) reported that *Daphnia* infochemicals (300 *D. magna* individuals/L grown for 24 h in the WC medium) generally had a weak influence on MC production in several *Microcystis* strains [38], while Becker (2010) demonstrated that when exposed to *Daphnia* medium (165 *D. magna* individuals/L grown in 24 h WC medium), *Microcystis* stopped producing MC [45]. In exposure to 2wDM our results showed similar findings: The concentration of intracellular MC-LR and des-MC-LR decreased significantly in MC+ compared with the control. Supposedly, in the high density DM (24 h) exposure, the concentration of both MC-LR and des-MC-LR decreased, owing to a lower cyanobacterial density being exposed to a higher number of *D. magna* producing more metabolites, which were not degraded during the short cultivation time. However, MC decrease could be due to its potential ability to bind to proteins (such as thioredoxin [62] and peroxiredoxin [34]) under stressful conditions [34], thus escaping detection. Further studies including either the expression of the genes involved in MC production or the detection of the protein-bound MC fraction are needed to clarify this. 

Hence, an overall decrease in the cyanobacterial physiology and the negative effect this *Daphnia* medium had on the toxic strain was demonstrated. 

To note, all our controls exhibited similar trends in dynamics where intracellular MC-LR increased on the third day and then decreased in the seven-day experiment. Similar results were observed by Becker 2010 [45], suggesting that this performance might not be caused by zooplankton. The transcription of *mcyHIJ* and *mcyB* genes increased in *M. aeruginosa* in 50% direct grazing treatments and in 90% of *Daphnia* infochemicals treatments after 24 h exposure, while transcription of *mcyACDEFG* was only expressed in 8% of the exposures [59]. The synthesis of MC, however, requires the expression of the whole MC synthetase cassette (*mcyABCDEFG*), therefore in that study zooplankton presence did not trigger MC production, however, it is yet to be clarified whether genes for these secondary metabolites synthesis need more time to respond than has been considered in this study [59]. *mcy A* was expressed in the first five days of *D. magna* exposure in the *M. aeruginosa* UTEX LB2385 strain, while in the Ch10 strain this occurred after 10 days, demonstrating a different response between strains [47]. As gene expression after stress vary in time depending on the particular gene and the strain, and moreover may or may not lead to MC production, we favored the analysis of the metabolite by chemical analysis as applied in this study over gene expression by RT qPCR. Additionally, it has been documented that *Microcystis* response to *Daphnia* medium is strongly strain specific [38], leaving the role of MC as anti-grazer compound arguable [28]. 

In addition to MC, *Microcystis* produces a range of peptides, such as cyanopeptolins, aeruginosins, and aerucyclamides that negatively influence digestive enzymes in zooplankton [56]. Metabolic pathways and triggers for production of these group of metabolites, as well as their functional role still needs more research [65]. Production of MC is energetically costly [66], allowing strains lacking MC to allocate energy in possible alternative mechanisms of defense or production of other secondary metabolites [34,58]. Our results show that the overall concentrations of CP A and AC D was higher in MC- then in MC+ in controls as well as the treatments. Interestingly, in monocultures, higher concentrations of cyanopeptolins, aerucyclamides, and aeruginosins were produced by the non-MC-producing mutant in comparison with the wild type [36]. When exposed to 1 wDM, concentrations of CP A and AC D did not change significantly in either toxic or nontoxic strain in our seven-day experiment. Similar results were observed in low density DM (2w) exposure in the nontoxic strain. The toxic strain, however, increased the export of CP A as a response to *Daphnia* medium, which might be a potential defensive mechanism. Cyanopeptolins are known as protease inhibitors [67] produced similarly to MC, through non-ribosomal pathways [49] with negative impact on zooplankton via inhibiting their trypsin-like digestion enzymes [6,56,68]. Thus, one of their potential roles could be grazing avoidance. If or not CP A has an anti-grazing effect or role will be proven in future studies. In order to understand fate and possible triggers for cyanopeptolins and aerucyclamides production, Sadler and von Elert (2014) [26] found increased production of CP B in *M. aeruginosa* PCC7806 against *D. magna* grazing, suggesting an induced defense mechanism, while relative and total amounts of CP A and C were not affected by *D. magna* presence. The transcription of genes encoding cyanopeptolins, microviridins, and aeruginosins in direct and indirect exposure to *D. magna* and *D. pulex* was mostly unaffected [59]. However, it is yet to be clarified whether genes for the synthesis of these secondary metabolites need more time to respond than was considered in this study (24 h) [59]. 

Different responses to *D. magna* spent medium in our experiment were strain dependent. MC- used in this study is a genetically engineered strain and its physiology as well as the response to *D. magna* spent medium might differ from MC non-producing strains that naturally occur in aquatic environments. Therefore, more studies are needed to show how *D. magna* spent medium would affect other MC producing and MC non-producing cyanobacterial species. 

## 4. Conclusions

To the best of our knowledge, this is the first study showing a lower physiological state of a MC+ strain when exposed to DM than its MC- mutant. *D. magna* spent medium of the highest *D. magna* density cultivated for the shortest time (high density DM (24 h)) provoked the strongest effect, which can be attributed to both high zooplankton density and a short time for bacterial degradation of the exudates. 

Photosynthetic activity as a proxy of physiological state of the MC+ strain was negatively affected by low density DM (2w) and high density DM (24 h). The mutant strain remained in good physiological state in all exposure scenarios, suggesting that it coped better with the stress induced by DM. The production of intracellular MC-LR, as well as other secondary metabolites in MC+ was decreased, as a consequence of the affected cell physiology, while the dynamics of the quantified metabolites were not affected in the mutant strain (cyanopeptolin A and aeruginosin D being higher in the MC- compared with MC+).

Extracellular cyanopeptolin A concentrations, however, increased in MC+ in the presence of 2wDM, suggesting a potential anti-grazing role, which is in line with its previously evidenced inhibition of trypsin-like digestion enzymes in *Daphnia* [52]. Furthermore, to the best of our knowledge, this is the first study showing that the concentration of extracellular CP A increased in MC+ when exposed to DM. The MC- strain did not show this increase in the treatment, compared to the control, possibly because the concentration was already initially that high. Therefore, more research is needed to confirm this role. In the presence of the tested *Daphnia* media, MC-LR and des-MC-LR do not appear to have a protective role for the strain, as their intracellular concentration decreased while the extracellular remained unchanged.

This study evidenced negative impact of *D. magna* spent medium on the MC+ strain, that was dependent on densities of the organisms in the seven days of exposure. 

## 5. Materials and Methods 

### 5.1. Culture Conditions

#### 5.1.1. Cyanobacterial Cultures

Two axenic strains were used in the experiments: MC+ and its mutant MC-, both obtained from the Pasteur Culture collection of Cyanobacteria [69]. MC- is MC+ transformed by inserting a chloramphenicol resistance cartridge into the *mcyB* gene involved in microcystin biosynthesis. This transformation led to the inability of derived mutant cells to produce any variant of microcystin [70], making MC- a useful tool for exploring the precise effects of microcystins on cyanobacterial physiology and metabolism, when compared to MC+ [70]. Before the start of the experiments, strains were cultivated in BG11 medium (SIGMA), with the addition of 5 µg chloramphenicol mL^−1^ for the MC-, to maintain the selection of the mutant cells. However, to exclude any impact of the chloramphenicol on DM, the experiments were performed without an antibiotic in the medium, as well as in the control medium. Both strains were grown under a 14 h:10 h light:dark regime using daylight white fluorescent tubes (Toshiba, 15 W, FL15D) with 20 μmol photons m^−2^ s^−1^ illumination at a constant temperature of 20 ± 1 °C (Sanyo incubator). Cultures were maintained in exponential growth phase by repeated dilution every 3 weeks in fresh culture medium, while axenicity was regularly evaluated as described in Reference [58]. 

#### 5.1.2. D. Magna

The *D. magna* clone (originating from the INERIS) was provided by D. Azam and M. Coke from the PEARL INRA 1036 U3E system (The National Infrastructure in Biology and Health, France). *D. magna* were slowly acclimatised during three weeks to the cyanobacterial BG11 medium that was used in all the experiments. The green algae *Scenedesmus communis* (isolated in our laboratory, originating from the lake of Grand Lieu) was provided by Bertrand Le Rouzic (University of Rennes 1). The animals were fed every second day with *Scenedesmus communis* (reaching max ≈ 2 × 10^4^ cells/mL in the aquarium at feeding time) grown in BG11 under the same conditions as the cyanobacteria. Some published studies showed that *Scenedesmus* has a negative impact on *Microcystis* through allopathic interactions [71,72]. In our experimental setup however, *Scenedesmus* have been used as *Daphnia* food during the medium preparations; therefore, *Daphnia* medium could contain *Scenedesmus* metabolites that could affect cyanobacteria [71,72]. However, *Scenedesmus* cell densities were very low when given to the *Daphnia* compared to those used in the above mentioned studies showing effects (strains mixed together using equal cell densities of 5 × 10^5^ cells/mL (*S. huji* with the *M. aeruginosa* PCC 7005) [72] and 1 × 10^5^ cells/mL (*S. acuminatus* with the *M. panniformis* BCCUSP200) [71]). In our experimental setup, *Scenedesmus* was given as the only food source, it was consumed by *Daphnia*, leaving not many, if any, *Scenedesmus* cells that could produce allopathic compounds affecting cyanobacteria. Noteworthy, when used in the experiment, *Daphnia* spent medium was mixed with cyanobacterial culture in 4:1 ration, therefore diluting even more possible *Scenedesmus* compounds. Hence it seems unlikely that the *Microcystis* strains were affected by *Scenedesmus* in this experimental setup. *D. magna* were cultivated at the constant temperature of 20 °C, light intensity of 15 μmol photons m^−2^ s^−1^ and a day/night cycle of 14 h/10 h (Sanyo incubator). 

### 5.2. Experimental Design

Experiments were performed in the three different exposure scenarios (Table 4), where MC+ and its mutant MC- were exposed to *D. magna* spent medium.

The preparation of the media for the experiments: In order to test environmentally relevant scenarios where *Daphnia* infochemicals are accumulated over time, we cultivated 75 neonatal individuals of *D. magna*/L for 1 week (low density DM (1w)). However, we observed no significant difference between control and treatment in this exposure in the photosynthetic activity (Figure S1) and metabolites dynamics (Figures S2 and S3), therefore 75 all age individuals of *D. magna* /L were cultivated for 2 weeks (low density DM (2w)). Moreover, we wanted to exclude potential degradation of *Daphnia* infochemicals, caused by bacteria coexisting in the *D. magna* medium during the 2-week cultivation period, as well as provoke a stronger effect in cyanobacteria. Therefore, we cultivated a high number of *Daphnia* (75 all age individuals of *D. magna* /L) for 24 h (high density DM (24 h)). One batch of spent medium was prepared per *Daphnia* treatment group. *D. magna* were cultivated in BG11 in aquariums and medium was filtered through 0.2 µm filter to remove bacteria and other particles and then stored at −20 °C. Before being used in experiments, exponentially growing cyanobacteria were centrifuged and cells were introduced in fresh BG11 medium and grown for one week to reach a cyanobacterial cell density of 1 × 10^7^ cells/mL for low density DM (1w) and low density DM (2w) exposures (pre-culture). Relative densities between cyanobacteria and low density DM (1w) and low density DM (2w) exposures did not provoke strong effects, therefore, the cyanobacterial density was lowered for the 24 h DM exposure, reaching 2x10^6^ cells/mL after one week. Filtered *D. magna* and fresh BG11 media for the treatments and for the controls, respectively, were mixed with the one week old cyanobacterial pre-cultures in 4:1 ratio in 2 L Erlenmeyer flasks. Cyanobacterial density at the beginning of the experiment was 2 × 10^6^ cells/mL for low density DM (1w and 2w) exposures and 5 × 10^5^ cells/mL for high density DM (24 h) exposure. 

The initial nutrients (PO_4_ and NO_3_) and pH were measured for the low density DM (2w) exposure to ensure similar conditions. Dissolved nutrient concentrations were measured from filtered (GF/F 0.7 µm) water using common colorimetric methods [73]. Nitrate was measured after reduction to nitrite on a cadmium-copper column [74]. Phosphate was measured following the method of Murphy and Riley (1962) [75]. Data presented in Table S1 show no significant difference between control and treatment (*p* > 0.05) for PO_4_ measurements in both strains, and for NO_3_ in the toxic strain. NO_3_ was significantly increased by 8% in the control of the MC- strain, which is still within the range of optimal growth conditions. We also measured pH that was not significantly different between control and treatment (*p* > 0.05) in all exposure scenarios (Table S2). Overall, there was no nutrient of pH differences that could affect the cyanobacteria physiology.

The procedure was the same for both strains, MC+ and its mutant MC-. The control and the treatment were done simultaneously. Experiments were done in triplicate, cultivated in 1 L flasks, and mixed every day for seven days. 

### 5.3. Photosynthetic Activity

To characterize the physiological state of the cyanobacteria, the electron transport rate (ETR) was measured every two days with a pulse-amplitude-modulated fluorescence monitoring system (PhytoPAM, Walz, Germany), following Reference [76] and [60]. The PhytoPAM is equivalent to four separate PAM-fluorometers using light-emitting diodes (LED) with 10 s light pulses at four different excitation wavelengths (470, 520, 645 and 665 nm), with the 645 nm specific to cyanobacteria (due to phycocyanin and allophycocyanin absorption). The PhytoPAM was used with only one channel, corresponding to the cyanobacteria. The reference excitation spectrum measured at the factory was used, as it was not significantly different from reference excitation spectra performed on our cyanobacterial cultures. After being dark adapted for 15 min, Ft (the instantaneous steady state fluorescence at every successive step of actinic irradiance) and Fm (the maximum fluorescence) were measured during a 20 increments of actinic irradiance from 1 to 1864 µmol photons m^−2^ s^−1^, with a 10 s time interval between successive steps. The ETR was calculated as follows: (Fm-Ft)/Fm x 0.42 x PAR (PAR: photosynthetic radiation) [60,76]. The ETRmax was inferred using the regression model of [77] to fit the ETR irradiance curves.

### 5.4. Cyanobacterial Cell Density

Cyanobacterial cell density was followed by measuring the optical density on a spectrophotometer (UVIKONxs SECOMAN) at a corresponding absorbance at 750 nm. Culture medium BG11 was used as reference (blank). The optical density was used to calculate the cyanobacterial cell density (cell number/mL) [58] in order to establish a relationship between the optical density at 750 nm and the cell density, calibration curves with *Microcystis* cell number/mL vs corresponding absorbance were established for both strains [60]. The counting of the cells was carried out using a Nageotte cell and observed under the Olympus BX50 microscope (objective 40). 

### 5.5. Analysis of Cyanobacterial Secondary Metabolites

The dynamics of intracellular and extracellular cyanobacterial peptides of MC+ and MC- in response to different *D. magna* media were analyzed using a Waters Acquity Ultra-High Performance Liquid Chromatography coupled to a photo diode array detector (PDA) and Xevo quadrupole time of flight mass spectrometer in series. Samples were centrifuged to separate cells (for intracellular metabolites) from supernatant (for extracellular metabolites) and lyophilized. Freeze-dried material was extracted in 0.5 ml 50% aqueous methanol. Extracts were separated on a CORTECS C18 column (0.2 mm ID × 100 mm long), which was maintained at 40 °C. The mobile phase was Milli-Q Water plus 0.1% formic acid (A) and acetonitrile plus 0.1% formic acid (B). Separation was achieved using a gradient increasing from 20% B to 70% B over 10 min, followed by a 100% B wash step and re-equilibration. Autosampler was maintained at 6 °C at all times. UV data was acquired from 200 to 400 nm. Mass data was acquired in positive ion electrospray scanning from *m*/*z* 50 to 2000 with a scan time of 2 s and inter-scan delay of 0.1 s. Ion source parameters, i.e., a capillary and sampling cone, were 2.9 V and 25 V respectively; desolvation temperature, 300 °C; and source temperature, 80 °C. Cone gas and desolvation gas flows were 50 L h^−1^ and 400 L h^−1^ respectively. Sodium iodide (2 μg μL^−1^ 211 in 50/50 Propan-2-ol/H2O) was used as the calibrant with leucine-enkephalin (0.5 mg mL^−1^ 212 in 50/50 methanol/Milli-Q) as the lock spray. Instrument control, data acquisition (centroid) and processing were achieved using MassLynx v4.1 software (Waters, Milford, MA, USA). We monitored secondary metabolites produced by *Microcystis* that were observed to be changing due to abiotic or biotic factors [36,50,78] (Table 5). Cyanobacterial peptides were detected using extracted ion chromatograms for the respective specific masses of the different compounds (Table 5).

MC-LR, des-MC-LR, cyanopeptolin A, and aerucyclamide D were quantified in our strains. Quantification was achieved using linear relationship between peak area (MC-LR and des-MC-LR at 238 nm, cyanopeptolin A at 220 nm, and aerucyclamide D at 240 nm) and known concentrations of the toxin standards. The microcystin-LR standard was purified as previously described [84]. Cyanopeptolin A standard and aerucyclamide D standard were purified using preparative HPLC (Biotage Parallex Flex, Cardiff, UK) and Flex V3 software for instrument control and data acquisition. The separation was performed on Atlantis Prep C18 column (5 µm particle size, 19 mm ID × 300 mm long; Waters, Elstree, UK) using a 30-min linear gradient from 60% to 100% methanol in MilliQ water. Absorbance was monitored at 210 nm and 280 nm. The flow rate was 20 mL/min and 4 mL fractions were collected. MassLynx v4.1 was used for both detection and quantification of the cyanobacterial peptides.

### 5.6. Statistics

R Core Team (2013) was used to access statistical analysis of the obtained data. All data are presented as mean ± standard deviation. Significant differences were determined at *p* < 0.05. We performed *t*-tests to determine the difference between photosynthetic activity of control and treatment for both strains (exposed to different DM (low density DM (2w) and high density DM (24 h)). T-test was also used to compare concentration of intracellular and extracellular metabolites in control and treatment for each strain. Additionally, one-way ANOVAs were used for the comparison between strains in the experiments. Prior to the ANOVAs, Shapiro–Wilk-Normality tests was performed. Where p value was >0.05, Tukey post-hoc test for pairwise comparison was performed (Control MC+/Control MC- and DM MC+/DM MC-). Where p value was <0.05 (Shapiro–Wilk-Normality tests), Kruskal–Wallis ANOVA was done, followed by t-tests for pairwise comparison (Control MC+/Control MC- and DM MC+/DM MC-).

## Figures and Tables

**Table 1:** Statistical comparison of photosynthetic activity between MC+ and MC- strains.

	Day 0	Day 1	Day 3	Day 5	Day 7
**Low Density DM (2w) Experiment**					
Control MC+/Control MC-	NS	*	NS	NS	NS
low density DM (2w) MC+/low density DM (2w) MC-	NS	NS	NS	**	***
Control MC+/low density DM (2w) MC+	+	+	++	NS	NS
Control MC-/low density DM (2w) MC-	NS	NS	NS	NS	***
**High Density DM (24 h) Experiment**					
Control MC+/Control MC-	NS	NS	NS	NS	NS
high density DM (24 h) MC+/high density DM (24 h) MC-	**	**	***	NS	¤¤
Control MC+/low density DM (24 h) MC+	NS	++	++	+++	++
Control MC-/low density DM (24 h) MC-	NS	NS	NS	NS	NS

* (*p* < 0.05), ** (*p* < 0.01), *** (*p* < 0.001) with Tukey post-hoc test after one way ANOVA; ¤¤ (*p* < 0.01) with t-test after Kruskal–Wallis; + (*p* < 0.05), ++ (*p* < 0.01), +++ (*p* < 0.001) with *t*-test; statistically non-significant (NS) (*p* > 0.05).

**Table 2:** Statistical comparison of intracellular and extracellular MC+ and MC- metabolites in low density DM (2w) exposure.

Low Density DM (2w) Experiment	Day 0	Day 3	Day 7	Day 0	Day 3	Day 7
	**CP A Intracellular**			**AC D Intracellular**		
Control MC+/Control MC-	NS	*	*	NS	**	*
low density DM (2w) MC+/ low density DM (2w) MC-	NS	**	NS	NS	NS	NS
Control MC+/ low density DM (2w) MC+	NS	++	NS	NS	++	NS
Control MC-/ low density DM (2w) MC-	NS	NS	NS	NS	NS	NS
	**CP A Extracellular**			**AC D Extracellular**		
Control MC+/Control MC-	NS	**	**	¤	**	NS
low density DM (2w) MC+/ low density DM (2w) MC-	**	**	NS	NS	***	NS
Control MC+/ low density DM (2w) MC+	NS	NS	+	NS	NS	NS
Control MC-/ low density DM (2w) MC-	NS	NS	NS	NS	NS	NS
	**MC-LR Intracellular**			**des-MC-LR Intracellular**		
Control MC+/ low density DM (2w) MC+	NS	++	NS	NS	++	NS
	MC-LR Extracellular			des-MC-LR Extracellular		
Control MC+/ low density DM (2w) MC+	NS	NS	NS	NS	NS	NS

* (*p* < 0.05), ** (*p* < 0.01), *** (*p* < 0.001) with Tukey post-hoc test after ANOVA; ¤ (*p* < 0.05) with t-test after Kruskal-Wallis; + (*p* < 0.05), ++ (*p* < 0.01) with t-test; NS (*p* > 0.05).

**Table 3:** Statistical comparison of intracellular and extracellular metabolites in low density DM (24 h) exposure.

High Density DM (24 h) Experiment	Day 0	Day 3	Day 7	Day 0	Day 3	Day 7
	**MC-LR Intracellular**			**des-MC-LR Intracellular**		
Control MC+/ low density DM (24 h) MC+	NS	++	NS	NS	++	++
	**MC-LR extracellular**			**des-MC-LR Extracellular**		
Control MC+/ low density DM (24 h) MC+	NS	NS	NS	NS	NS	+

+ (*p* < 0.05), ++ (*p* < 0.01) with *t*-test; NS (*p* > 0.05).

**Table 4:** *D. magna* spent medium characteristics and cyanobacterial cell densities used in the experiments.

*D. magna* Spent Medium Characteristics (One Batch of DM per *Daphnia* Treatment Group)	Cyanobacterial Cell Density After 1 Week Pre-Cultivation for MC+ and MC-	Cyanobacterial Cell Density at the Beginning of the Experiment for MC+ and MC-
75 neonatal individuals of *D. magna*/L cultivated for 1 week (low density DM (1w))	1 × 10^7^ cells/mL	2 × 10^6^ cells/mL
75 all age individuals of *D. magna*/L cultivated for 2 weeks (low density DM (2w))	1 × 10^7^ cells/mL	2 × 10^6^ cells/mL
200 all age individuals of *D. magna*/L cultivated for 24 h (high density DM (24 h))	2 × 10^6^ cells/mL	5 × 10^5^ cells/mL

**Table 5:** Secondary metabolites produced in *M. aeruginosa* PCC7806.

Peptide Class	Peptide Sub-Class	*m/z* [M+H]+	References
Microcystins			
	MC-LR	996	[79]
	Des-MCLR	982	[79]
Cyanopeptolins (CP)			
	CP 963A	947	[51]
	CP A	958	[80]
	CP B	930	[80]
Aerucyclamides (AC)			
	AC C	517	[81]
	AC B	533	[82]
	AC A	535	[82]
	AC D	587	[81]
Aeruginosins			
	Aeruginosin 684	685	[36]
	Aeruginosin 602	603	[83]

## Supplementary Materials

The following are available online at https://www.mdpi.com/2072-6651/11/7/421/s1, Figure S1: Photosynthetic activity of MC- and MC+ when cultured in *D. magna* spent medium (DM) and cultured in BG11 (Control): (**A**) low density DM (1w) MC- (**B**) low density DM (1w) MC-, Figure S2: Dynamics of intracellular and extracellular metabolites in MC+ when exposed to low density DM (1w) compared to when grown in BG11 (Control): (**A**) intracellular MC-LR, (**B**) intracellular des-MC-LR, (**C**) extracellular MC-LR, (**D**) extracellular des-MC-LR. (*p* > 0.05) *t*-test, Figure S3: Dynamics of intracellular and extracellular metabolites in MC+ and MC- when exposed to low density DM (1w): *M. aeruginosa* PCC7806 (low density DM (1w) MC+) and *M. aeruginosa* PCC7806 *mcy-* (low density DM (1w) MC-) and when grown in BG11: *M. aeruginosa* PCC7806 (Control MC+) and *M. aeruginosa* PCC7806 *mcy-* (Control MC-). (**A**) intracellular CP A, (**B**) intracellular AC D, (**C**) extracellular CP A, (**D**) extracellular AC D. (*p* > 0.05) *t*-test, Table S1: Concentration of PO4 (mg/L) and NO3 (mg/L) on day 0 in the 2wDM experiment, Table S2: pH on day 0 in the 1wDM, 2wDM and 24 h DM.

## Abbreviations

MC+	*M. aeruginosa* PCC7806
MC-	*M. aeruginosa* PCC7806 mcy-
DM	*Daphnia* medium
low density DM (1w)	spent medium in which 75 neonatal individuals of *D. magna*/L were cultured for 1 week
low density DM (2w)	spent medium in which 75 all age individuals of *D. magna*/L were cultured for 2 weeks
high density DM (24 h)	spent medium in which 200 all age individuals of *D.magna*/L were cultured for 24 h
MC-LR	microcystin LR
des-MC-LR	des microcystin LR
CP	cyanopeptolin
AC	aerucyclamide
CP A	cyanopeptolin A
AC D	aerucyclamide D
Control MC+	*M. aeruginosa* PCC7806 cultivated in BG11
Control MC-	*M. aeruginosa* PCC7806 mcy- cultivated in BG11
