# Supplementary material for: Daphnia magna Exudates Impact Physiological and Metabolic Changes in Microcystis aeruginosa
Source: Toxins (Basel). 2019 Jul 19;11(7):421. doi: 10.3390/toxins11070421 (PMC6669642; doi:10.3390/toxins11070421)
Supplement: Supplementary file 1 [file toxins-11-00421-s001.pdf]

Supplementary Materials: *Daphnia magna* Exudates Impact Physiological and Metabolic Changes in *Microcystis aeruginosa*

Gorenka Bojadzija Savic, Christine Edwards, Enora Briand, Linda Lawton, Claudia Wiegand and Myriam Bormans

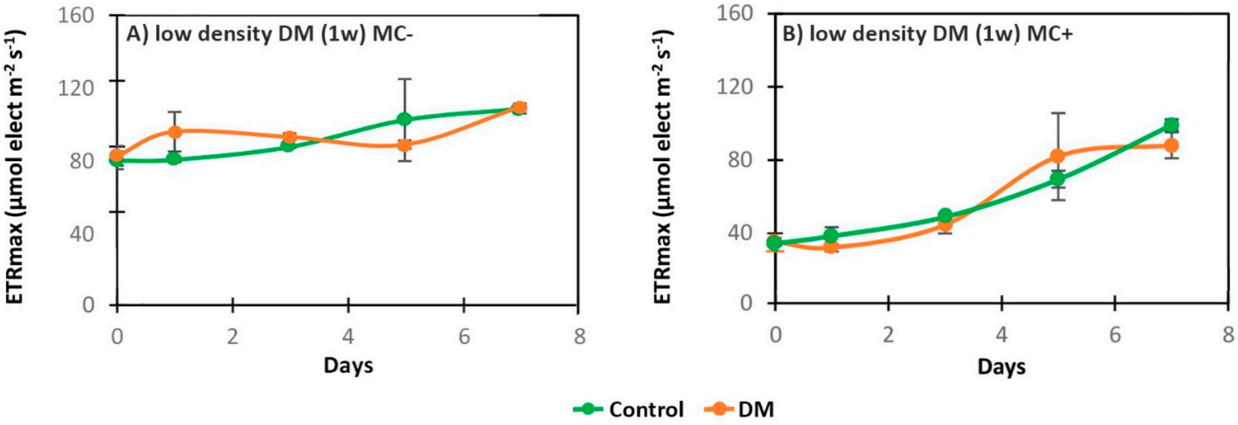

Figure S1. Photosynthetic activity of MC- and MC+ when cultured in *D. magna* spent medium (DM) and cultured in BG11 (Control): (**A**) low density DM (1w) MC- (**B**) low density DM (1w) MC-.

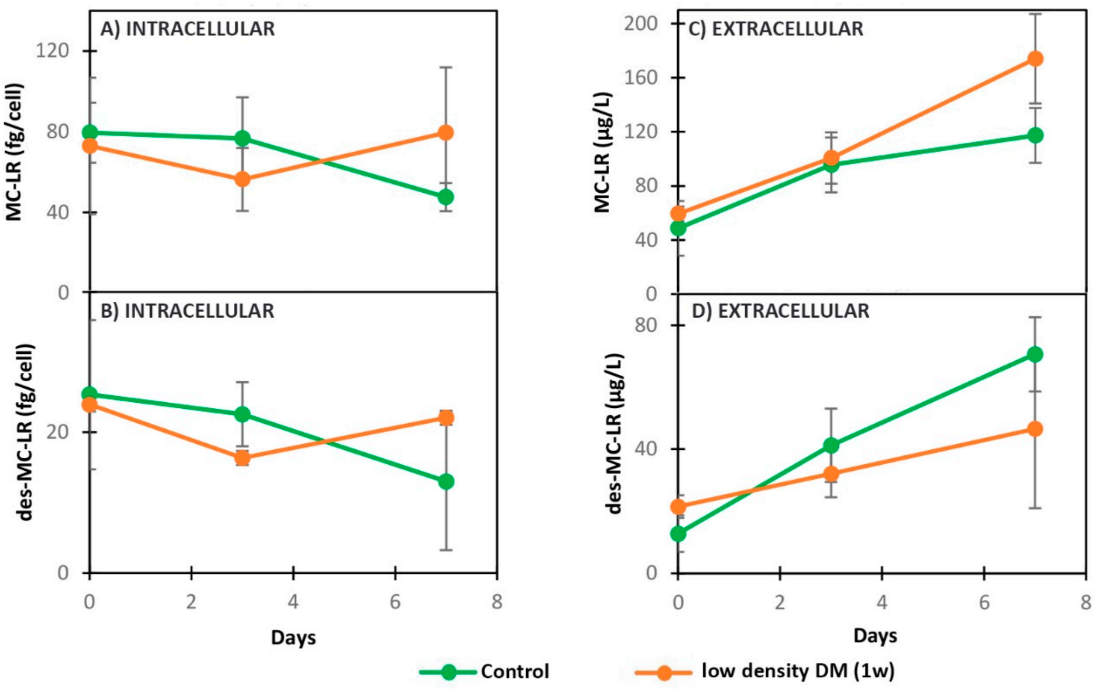

Figure S2. Dynamics of intracellular and extracellular metabolites in MC+ when exposed to low density DM (1w) compared to when grown in BG11 (Control): (**A**) intracellular MC-LR, (**B**) intracellular des-MC-LR, (**C**) extracellular MC-LR, (**D**) extracellular des-MC-LR. ($p > 0.05$) *t*-test.

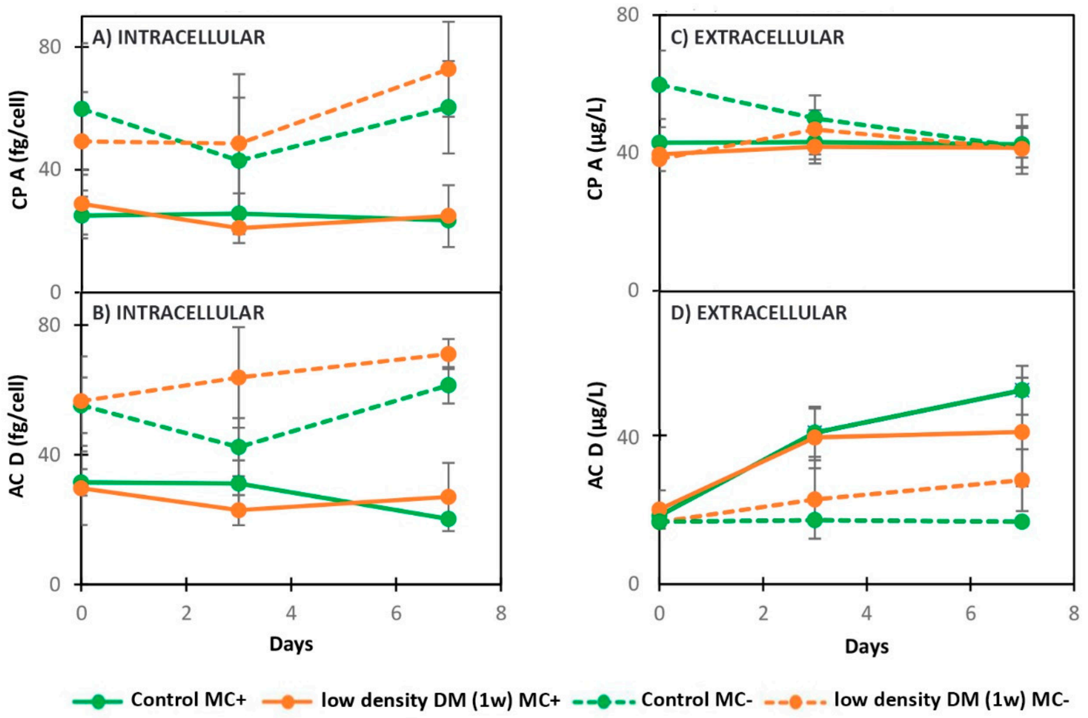

Figure S3. Dynamics of intracellular and extracellular metabolites in MC+ and MC- when exposed to low density DM (1w): *M. aeruginosa* PCC7806 (low density DM (1w) MC+) and *M. aeruginosa* PCC7806 *mcy-* (low density DM (1w) MC-) and when grown in BG11: *M. aeruginosa* PCC7806 (Control MC+) and *M. aeruginosa* PCC7806 *mcy-* (Control MC-). (**A**) intracellular CP A, (**B**) intracellular AC D, (**C**) extracellular CP A, (**D**) extracellular AC D. ($p > 0.05$) *t*-test.

Table S1. Concentration of PO4 (mg/L) and NO3 (mg/L) on the day 0 in the 2wDM experiment.

	**Day 0**	
	PO4 (mg/L)	NO3 (mg/L)
2wDM MC+ Control	4.20 ± 0.35	173.09 ± 25.43
2wDM MC+ Treatment	4.16 ± 0.15	200.66 ± 22.36
2wDM MC- Control	4.50 ± 0.09	248.46 ± 3.81
2wDM MC- Treatment	4.24 ± 0.17	232.85 ± 2.46

Table S2. pH on the day 0 in the 1wDM, 2wDM and 24 h DM.

**Day 0**	
	pH
1wDM MC+ Control	8.20 ± 0.10
1wDM MC+ Treatment	8.23 ± 0.16
1wDM MC- Control	8.19 ± 0.02
1wDM MC- Treatment	8.24 ± 0.04
2wDM MC+ Control	8.19 ± 0.02
2wDM MC+ Treatment	8.25 ± 0.08
2wDM MC- Control	8.14 ± 0.05
2wDM MC- Treatment	8.18 ± 0.08
24hDM MC+ Control	8.17 ± 0.06
24hDM MC+ Treatment	8.18 ± 0.08
24hDM MC- Control	8.18 ± 0.02
24hDM MC- Treatment	8.17 ± 0.06
